# *In Vivo* Analysis of MEF2 Transcription Factors in Synapse Regulation and Neuronal Survival

**DOI:** 10.1371/journal.pone.0034863

**Published:** 2012-04-09

**Authors:** M. Waseem Akhtar, Mi-Sung Kim, Megumi Adachi, Michael J. Morris, Xiaoxia Qi, James A. Richardson, Rhonda Bassel-Duby, Eric N. Olson, Ege T. Kavalali, Lisa M. Monteggia

**Affiliations:** 1 Department of Psychiatry, University of Texas Southwestern Medical Center, Dallas, Texas, United States of America; 2 Department of Molecular Biology, University of Texas Southwestern Medical Center, Dallas, Texas, United States of America; 3 Department of Pathology, University of Texas Southwestern Medical Center, Dallas, Texas, United States of America; 4 Department of Neuroscience, University of Texas Southwestern Medical Center, Dallas, Texas, United States of America; 5 Department of Neuroscience, University of Texas Southwestern Medical Center, Dallas, Texas, United States of America; University of Insubria, Italy

## Abstract

MEF2 (A–D) transcription factors govern development, differentiation and maintenance of various cell types including neurons. The role of MEF2 isoforms in the brain has been studied using *in vitro* manipulations with only MEF2C examined *in vivo*. In order to understand specific as well as redundant roles of the MEF2 isoforms, we generated brain-specific deletion of MEF2A and found that *Mef2aKO* mice show normal behavior in a range of paradigms including learning and memory. We next generated *Mef2a* and *Mef2d* brain-specific double KO (*Mef2a/d*DKO) mice and observed deficits in motor coordination and enhanced hippocampal short-term synaptic plasticity, however there were no alterations in learning and memory, Schaffer collateral pathway long-term potentiation, or the number of dendritic spines. Since previous work has established a critical role for MEF2C in hippocampal plasticity, we generated a *Mef2a*, *Mef2c* and *Mef2d* brain-specific triple KO (*Mef2a/c/d*TKO). *Mef2a/c/d* TKO mice have early postnatal lethality with increased neuronal apoptosis, indicative of a redundant role for the MEF2 factors in neuronal survival. We examined synaptic plasticity in the intact neurons in the *Mef2a/c/d* TKO mice and found significant impairments in short-term synaptic plasticity suggesting that MEF2C is the major isoform involved in hippocampal synaptic function. Collectively, these data highlight the key *in vivo* role of MEF2C isoform in the brain and suggest that MEF2A and MEF2D have only subtle roles in regulating hippocampal synaptic function.

## Introduction

Myocyte enhancer factor 2 (MEF2) transcription factors regulate development of various tissue types, including muscle (cardiac, smooth, and striated), bone, and lymphocytes [1]. While members of the MEF2 family (MEF2A to MEF2D) are critical during muscle differentiation and cardiovascular function, their individual roles within the central nervous system are largely unknown. In the mouse brain, *Mef2a*, *c* and *d* are expressed at high levels in multiple regions, including cortex, hippocampus and cerebellum [2]. Distinct patterns of expression during pre- and postnatal development suggest specific and dissociable functions for each MEF2 protein at different stages of neuronal maturation.

Inhibition of MEF2 function in cultured cortical neurons has been shown to cause apoptotic cell death, suggesting that MEF2-dependent transcriptional regulation is necessary for neuronal survival [3]. Moreover, *in vitro* genome-wide analysis of MEF2 transcriptional activity identified several activity-dependent MEF2 target genes that regulate a variety of aspects of synaptic function, including excitatory synapse weakening, excitatory synapse maturation, inhibitory synapse development, and presynaptic vesicle release [4]. Recent studies have started to focus on the role of individual MEF2 isoforms in neuronal function *in vitro*. RNA-interference-mediated knockdown of both MEF2A and D in hippocampal cultures has been shown to increase excitatory synapse number and synaptic transmission [5]. Similar knockdown of MEF2A in cerebellar granule cells in organotypic cultures resulted in a decrease in the number of dendritic claws [6]. While these *in vitro* data strongly suggest important roles for specific MEF2 isoforms in neuronal function, the precise functions of the individual MEF2 isoforms *in vivo* remains to be defined. A previous study from our group showed that conditional deletion of *Mef2c* in brain results in a marked increase in the number of excitatory synapses, accompanied by potentiation of basal and evoked synaptic transmission, but with significant impairments in hippocampal-dependent learning and memory [7]. In a separate study, loss of *Mef2c* in nestin-expressing neural stem/progenitor cells was shown to impair neuronal differentiation *in vivo*, and play an important role in normal neuronal development, distribution, and synaptic activity in the cortex [8]. Collectively, the *in vivo* data suggests that MEF2C plays an important role in learning and memory, synaptic plasticity, and control of synapse number.

To examine the role of MEF2A in brain and examine the impact on behavior, we generated *Mef2a* brain-specific knockout (KO) (*Mef2a* ^KO^) mice. We found that deletion of *Mef2a* in the brain of mice results in no apparent abnormalities in a variety of behavioral tests, including learning and memory. Since earlier *in vitro* studies concluded that both MEF2A and D negatively regulate excitatory synapse number, we generated a *Mef2a* and *Mef2d* brain-specific double KO (*Mef2a/d* ^DKO^). Brain-specific deletion of both *Mef2a* and *Mef2d* caused deficits in motor coordination and enhanced short-term synaptic plasticity, but surprisingly did not impact fear conditioned associative learning, long-term potentiation, or synapse number. To more closely examine the contribution of the MEF2C isoform in the brain, we generated a *Mef2a*, *Mef2c* and *Mef2d* brain-specific triple KO (*Mef2a/c/d* ^TKO^). Mice with a triple deletion of *Mef2a*, *Mef2c*, and *Mef2d* in the brain show early postnatal lethality accompanied by increased neuronal apoptosis, suggesting redundant roles of MEF2 transcription factors in the regulation of neuronal survival. Moreover, *Mef2a/c/d* ^TKO^ mice show impairments in measurements of hippocampal short-term plasticity. These data suggest that MEF2A, C and D act redundantly in neuronal survival, but that MEF2C is the isoform involved in hippocampal synaptic function. These data contribute to an understanding of the role of the MEF2 transcription factors in learning and memory as well as synaptic function, especially considering the use of double and triple knockouts, which begin to resolve issues of redundancy and compensation.

## Materials and Methods

### Ethics Statement

All animal experimental procedures were reviewed and approved (APN 2008-0129) by the Institutional Animal Care and Use Committee at University of Texas Southwestern Medical Center.

### Generation of MEF2A conditional mice

Genomic regions of the *Mef2a* locus were isolated from 129SvEv genomic DNA by high-fidelity PCR (Takara LA taq PCR system) and cloned into a pGKneoF2L2DTA vector, which contains a neomycin resistance gene, flanked by FRT and *loxP* sites, and a diphtheria toxin gene cassette. A 1.7-kb genomic sequence (5′ arm) upstream of *Mef2a* exon 2 was cloned upstream of a 5′ loxP sequence in the pGKneoF2L2DTA vector. A 1.1-kb fragment (knockout arm) harboring exon2 was cloned between the 5′ loxP sequence and 5′ FRT sequence. A 3.7-kb fragment (3′ arm) downstream of *Mef2a* exon 2 was cloned downstream of the 3′ loxP sequence. The targeting vector was linearized with PvuI and electroporated into 129SvEv-derived ES cells. One thousand ES clones were isolated and analyzed for homologous recombination by PCR. One clone with a properly targeted *Mef2a* allele was injected into 3.5-day old C57BL/6 blastocysts, and the resulting chimera were crossed to C57BL/6 mice to achieve germline transmission.

**Figure 1:**
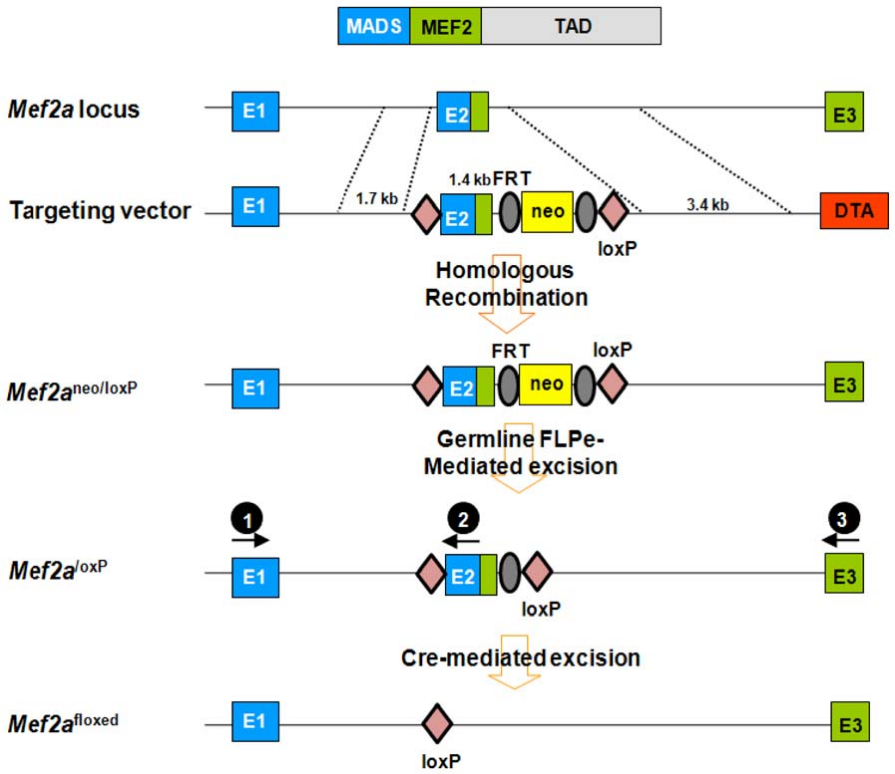
Strategy to generate a conditional MEF2A allele. LoxP sites were inserted into introns 1 and 2 through homologous recombination. Cre-mediated excision results in one loxP site in the place of exon 2.

To genotype the mice, tail genomic DNA was isolated using the HotSHOT methodology. The 25 µl PCR genotyping reaction contained 2 µl of tail DNA as template, 2.5 U of Taq polymerase (Promega), 1.5 µl of 10 µM primers (SA-fwd, KO-rev, LA-rev in mix; for sequences see below), 1 µl of 10 mM dNTPs and a final concentration of 2 mM MgCl_2_. An annealing temperature of 60°C was used and the PCR products were electrophoresed on a 2% agarose gel. The DNA sequences for the PCR genotyping primers, which are designated as black balls for location on the gene in Figure 1, are as follows: <Black ball 1> Gtm2ACKO-SA-fwd, 5′-GGTAGCTCAGGTGTCACTTCTTG-3′; <Black ball 2> Gtm2ACKO-KO-rev, 5′-CACTTTACATCCCAATAGCAGCC-3′; <Black ball 3> Gtm2ACKO-LA-rev, 5′-CTCATCCATTTATGGCTGTGTC-3′. The primers were added together into the reaction. The sizes of the PCR products are, WT band is 267 base pairs (bp); loxP band is 367 bp; and KO band is 500 bp.

### Histology

Mice were perfused and brains were post-fixed overnight in DEPC-treated 4% paraformaldehyde. The brains were paraffin-embedded, sectioned at 5 µm, and mounted on coated slides. Sections were stained with Nissl or Hematoxylin and eosin using standard procedures. For *in situ* hybridization, riboprobes were labeled with [α-^35^S]-UTP using the MAXIscript *in vitro* transcription kit (Ambion) following the manufacturer's instructions. *In situ* hybridization of sectioned tissues was performed as previously described [9]. TUNEL assay was performed according to the manufacturer's instructions (Roche). TUNEL positive cells were counted on at least three levels of coronal-section brain, and averaged from two 4 week-old animals for each genotype group. Data was presented as mean ± SEM, and student's t-test was used to analyze data with significance set as P<0.05.

### RNA isolation and quantitative RT-PCR

RNA was isolated from 4 week old animals (N=3 for each genotype). RNA was extracted from cortex, hippocampus and cerebellum using the RNeasy kit (Qiagen) following the manufacturer's instructions. Two µg of RNA was converted to cDNA using random primers and Superscript III reverse transcriptase (Invitrogen). Quantitative analysis was performed by real-time PCR using the ABI PRISM 7000 sequence detection system with TaqMan primers (Applied Biosystems) or with SYBR Green Master Mix reagent (Applied Biosystems). The fold change in RNA was calculated using the comparative Ct method, normalizing to GAPDH control. The primer sequences used for RT-PCR are as follows:

Mef2a qPCR forward, 5′-AACCGACAGGTTACTTTTAC-3′; Mef2a qPCR reverse, 5′-TCTTAACGTCTCAACGATAT-3′. To assess MEF2B, C & D, Taqman probes (ABI) were used: Mef2b, Mm00484953_g1; Mef2c, Mm01344728_m1; Mef2d, Mm00504931_m1; Gapdh, Mm99999915_g1.

### Golgi Staining

Golgi staining was performed using the FD Rapid GolgiStain Kit (FD Neuro Technologies) following the manufacturer's instructions. Briefly, animals were deeply anesthetized and brains were removed from the skull and rinsed in double distilled water. Brains were immersed in the impregnation solution, and stored at room temperature for 2 weeks in the dark. The impregnation solutions were replaced on the next day. Brains were transferred into Solution C and stored at 4°C for 1 week in the dark. Solution C was replaced the next day. Brain sections (100 µm thickness) were cut on a cryostat (Leica 430) with the chamber temperature set at -22°C. Each section was mounted with Solution C on saline-coated microscope slides. After absorption of excess solution, sections were naturally dried at room temperature. Dried sections were processed following the manufacturer's instructions. Quantification of dendritic spine density was performed as previously described [7]. Briefly, images of dendrites within the CA1 subregion of the hippocampus were made by using a ×63 objective with a Leica DM2000 microscope and an Optronics Microfire CCD camera. Spines were counted along CA1 secondary dendrites starting from their point of origin from the primary dendrite. A dendritic region in focus was traced to measure its length using ImageJ software. The number of spines on that region was then manually quantified by a blinded counter and divided by the region length to obtain spine density.

### Behavioral Overview

Mice were on a 12-hour light/dark cycle with *ad libitum* food and water. Behavioral tests were performed on adult male mice at least 2 months old comparing the specific KO line with wild type littermate control mice. Prior to all testing, mice were allowed to habituate in the behavioral room for one hour. The experimenters were blinded to the genotype in all tests. For all experiments, significance was P<0.05.

### Locomotor Activity

Animal were placed in a fresh home cage and locomotor activity was measured for 2 hours by photocell beams linked to computer data acquisition software (San Diego Instruments). Data were analyzed with repeated analysis of variance (ANOVA). Data were presented as mean ± SEM.

### Rotarod

Each mouse was placed on the rotarod (IITC Life Science). The rotarod was activated and its speed ramped up from 0–45 revolutions per minute in 60 seconds. The time to fall off the rotarod or turn one full revolution was measured for four consecutive trials. The mouse was returned to its original cage and 24 hours later the mice were tested for consecutive trials. Data were analyzed with repeated analysis of variance (ANOVA). Data were presented as mean ± SEM.

### Open-Field

Mice were placed in a 42 cm square open field under dim lighting and their activity was monitored for 6 minutes with a video tracking system using Ethovision software (Noldus). The time spent in the center of the open field, the periphery area, and the non-periphery area that is defined, as the area between the center and periphery was determined. Data was presented as mean ± SEM, and student's t-test was used to analyzed data with significance set as p<0.05.

### Fear Conditioning

The fear conditioning paradigm was assessed as previously described [10]. Briefly, mice were placed in individual chambers for two minutes followed by a loud tone (90 dB) for 30 sec, then immediately followed by a 0.5 mA footshock for 2 seconds. Mice remained in the box for one minute at which time they again received a loud tone (90 dB) for 30 sec and then an immediate 0.5 mA footshock for 2 seconds. The mice were immediately removed and placed back into their home cages. Each chamber was cleaned with 70% ethanol between animals. To test for context-dependent fear conditioning, 24 hours later the mice were placed back in the same boxes without a tone or shock. The amount of time the animal spent freezing was scored by an observer blind to the genotype. Freezing behavior was defined as no movement except for respiration. Four hours later, the cue test was performed. To test for cue-dependent fear conditioning, mice were placed in a novel environment with no tone or shock for three minutes followed by three minutes of the tone. The amount of time the mice spent freezing was then determined. Data was presented as mean ± SEM, and student's t-test was used to analyzed data with significance set as p<0.05.

### Slice Electrophysiology

For extracellular field recordings, 2–5 month old mice were used. Mice were anaesthetized by intraperitoneal nembutal injection and decapitated. The brain was rapidly dissected and hippocampal slices (400 µm) were collected in ice-cold dissection buffer containing in mM: 212 sucrose, 3 KCl, 5 MgCl_2_, 0.5 CaCl_2_, 1 NaH_2_PO_4,_ 26 NaHCO_3_, and 10 glucose. The CA3 region was cut to avoid epileptiform activity. Slices were placed at 30°C for 2 h in artificial CSF (ACSF) containing in mM: 124 NaCl, 5 KCl, 26 NaHCO_3_, 1.25 NaH_2_PO_4_, 2 CaCl_2_, 1 MgCl_2_, and 10 glucose. ACSF and dissection buffer were bubbled with 95% O_2_/5% CO_2_. For recording, slices were placed in a submersion-recording chamber, maintained at 30°C, and perfused with ACSF. Concentric, bipolar tungsten electrodes were used to activate Schaffer collateral/commissural (SC) fibers in the hippocampal CA1 region. Extracellular glass microelectrodes filled with ACSF (resistance, 1 MΩ) were placed in the stratum radiatum to measure field excitatory post-synaptic potentials (fEPSPs). For baseline recordings, slices were stimulated at 0.033 Hz for 20 min at stimulation intensities of 40%–50% of the highest measured fEPSP size. LTP was induced by applying a theta burst stimulus (TBS) consisting of three trains (10 s interval), with each train composed of 5 bursts (100 Hz). PPF was tested by applying two pulses with interstimulus intervals (ISIs) ranging from 20 ms to 500 ms. Data were sampled at 5 kHz and analyzed using a program written in LabView (National Instruments). PPF was analyzed by unpaired *t*-test. Input-output slopes were fit by linear regression and between group slopes were subsequently compared using unpaired *t*-tests. LTP was analyzed by two-way ANOVA with repeated measures to test the time X genotype interaction. Following a statistically significant interaction effect, Bonferroni-corrected *t*-tests were used for post-hoc analyses.

### Cell culture

Primary hippocampal neurons were cultured as previously described [11]. Briefly, whole hippocampi were isolated from floxed MEF2AD mice on postnatal days 0–1. After dissection, tissues were trypsinized for 10 min at 37°C, mechanically dissociated using siliconized glass pipettes, and plated onto Matrigel-coated coverslips. A concentration of 4 µM cytosine arabinoside (Sigma) was added at 1 day *in vitro* (DIV) and removed at 4 DIV. Lentivirus was generated as previously described [12], [13], [14]. Briefly, lentivirus containing either GFP or Cre was infected at 4 DIV and recordings were done at 15–18 DIV.

## Results

### Generation of brain-specific Mef2a knockout mice

**Figure 2:**
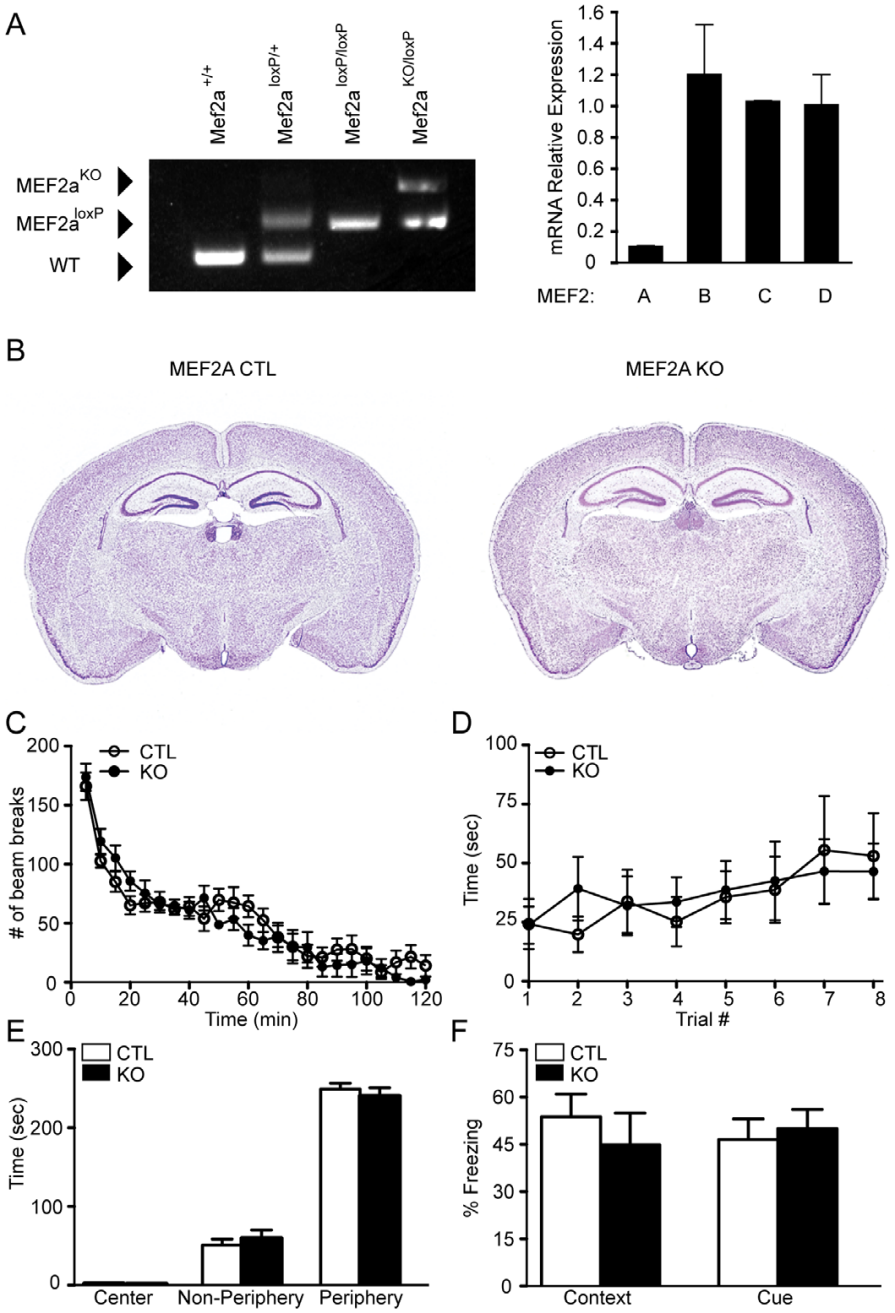
Mef2a^KO^ mice show normal behavior. (**A**) Left, PCR genotyping to distinguish different Mef2a alleles. Right, the bar graph shows expression levels of Mef2 isoforms in the hippocampus of 4 week old animals (n=3/genotype), detected by quantitative PCR. The fold change in RNA was calculated using the comparative Ct method, normalizing to GAPDH as a control. (**B**) Nissl staining of brain cross-sections from littermate control (CTL) and *Mef2a* ^KO^ mice at 2 months of age. Brain-specific deletion of MEF2A was achieved by crossing *MEF2A^loxP/loxP^* mice with mice harboring a transgene for *hGFAP-Cre.* (**C**) *Mef2a^KO^* (KO) mice show no significant differences in locomotor activity compared to littermate CTL mice as assessed by consecutive beam breaks over a two-hour period, although the number of beam breaks significantly decreased over time (F_23,184_=41.84, P<0.0001), there was no significant interaction between group and time (CTL, n=8; *Mef2a^KO^* mice, n=9). (**D**) *Mef2a^KO^* mice have no difference in motor coordination as assessed in the rotarod test compared to littermate CTL mice (CTL, n=8; *Mef2a^KO^* mice, n=9). (**E**) *Mef2a^KO^* mice exhibit normal anxiety-like behavior, as assessed in the open field test (CTL, n=8; *Mef2a^KO^* mice, n=9). (**F**) Context- and cue-dependent fear conditioning is unaltered in *Mef2a^KO^* mice relative to littermate CTL mice (CTL, n=8; *Mef2a^KO^* mice, n=9). All data is shown as mean ± SEM.

Mice homozygous for a *Mef2a* null mutation die suddenly within the first week of life due to defects in cardiac function [15], precluding any analysis of potential functions of *Mef2a* in the adult brain. Therefore, we generated a conditional *Mef2a* null allele with loxP sites (*Mef2a^loxP^*) in the introns flanking the second coding exon containing the MADS-box and MEF2 domain, which together mediate DNA binding, dimerization and cofactor interactions (Fig. 1). Deletion of the genomic region between the loxP sites inactivates the *Mef2a* gene [15](Fig. 2A, left). Mice homozygous for the *Mef2a^loxP^* allele showed no apparent abnormalities. *Mef2a* was then deleted specifically in the central nervous system (CNS) by breeding *Mef2a^loxP/loxP^* mice with transgenic mice that express Cre recombinase under the control of the human GFAP promoter (hGFAP-Cre), expressed in neuronal progenitor cells during late embryogenesis [16]. In hGFAP-Cre mice, Cre recombinase has been shown to express in nearly all CNS neurons and many oligodendrocytes, with the notable exceptions of Purkinje cells in the cerebellum, mitral cells in olfactory bulbs, and spinal motor neurons [16]. Our group had previously used the hGFAP-Cre line to delete MEF2C in brain and assess in vivo function [7]. Mice with homozygous deletion of *Mef2a* in the brain (*MEF2a^KO^*) were viable, fertile, and reached the same age and body weights as their control littermates. Deletion of the *Mef2a^loxP^* allele in the brains of mutant mice was confirmed by real-time PCR (Fig. 2A, right). Histological analysis by Nissl staining of brain sections revealed no obvious abnormalities in number or density of neurons in various brain regions (Fig. 2B).

### Brain-specific deletion of MEF2A does not affect behavior

We explored the potential involvement of *Mef2a* in behavior by testing the *MEF2a^KO^* mice in several behavioral paradigms. We found that *MEF2a^KO^* mice exhibited no apparent abnormalities in locomotor activity relative to wild type littermate controls (CTL) (Fig. 2C). We assessed motor coordination by rotorod testing and found no difference in the time the *MEF2a^KO^* mice spent on the rotarod compared to CTL mice (Fig. 2D). To investigate anxiety-related behavior, we used the open field test and found the *MEF2a^KO^* mice were indistinguishable from CTL animals (Fig. 2E). The *MEF2a^KO^* mice were examined in the fear-conditioning paradigm for learning and memory, as previously described for the *MEF2c^KO^* mice [7]. The baseline level of freezing assessed before training was indistinguishable in the *MEF2a^KO^* and CTL mice (data not shown). In the context- and cue-dependent fear conditioning, *MEF2a^KO^* mice showed no difference in time spent freezing compared to CTL mice (Fig. 2F), and there was no difference in the response of the *MEF2a^KO^* mice and CTL mice to footshock over a range of shock intensities (data not shown). These results suggest that MEF2A alone is not involved in the regulation of various behaviors, including learning and memory.

### Brain-specific deletion of both MEF2A and MEF2D affects motor coordination

**Figure 3:**
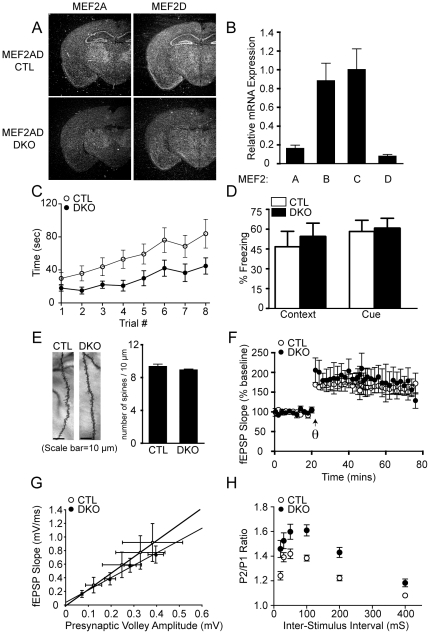
Brain-specific deletion of *Mef2a* and *Mef2d* causes impairments in motor coordination, as well as presynaptic release probability. (**A**) Detection of *Mef2a* and *Mef2d* transcripts by *in situ* hybridization in littermate CTL and *Mef2a/d* ^DKO^ (DKO) mice. Arrow indicates hippocampus. (**B**) Expression level of MEF2 transcription factors in the hippocampus of *Mef2a/d* ^DKO^ mice as detected by quantitative RT-PCR. RNA was isolated from the hippocampus of 4 week old animals (n=3/genotype), and then expression determined by quantitative PCR. The fold change in RNA was calculated using the comparative Ct method, normalizing to GAPDH as a control. (**C**) *Mef2a/d* ^DKO^ mice exhibit impaired motor coordination as assessed by falling off the accelerating rotarod faster than littermate CTLs (F_1,7_=27.64, P<0.05; CTL, n=8; *Mef2a/d* ^DKO^, n=10), posthoc analysis demonstrated that trial numbers 3–8 were significantly different between the *Mef2a/d* ^DKO^ and CTL mice. (**D**) *Mef2a/d* ^DKO^ mice show normal associative learning, as assessed by context- and cue-dependent fear conditioning (CTL, n=8; *Mef2a/d* ^DKO^, n=10). (**E**) Representative images of Golgi-stained CA1 pyramidal neurons and a bar graph comparing the number of dendritic spines per 10 µm length of dendrite from littermate CTL and *Mef2a/d* ^DKO^ mice (n=31 dendritic segments from 31 neurons of two CTL mice and n=68 dendritic segments from 68 neurons of four *Mef2a/d* ^DKO^ mice). (**F**) CA1 LTP induction and maintenance is normal in DKO mice (CTL, n=3; *Mef2a/d* ^DKO^, n=3). (**G**) Input-output relations are normal in *Mef2a/d* ^DKO^ mice (CTL, n=4; *Mef2a/d* ^DKO^, n=4; solid lines indicate lines of best fit.). (**H**) Paired-pulse facilitation is increased in *Mef2a/d* ^DKO^ mice at Inter-Stimulus Interval 20, 30, 50, 100, 200 and 400 (mS) compared to littermate CTLs, suggesting decreased presynaptic release probability (CTL, n=8; *Mef2a/d* ^DKO^, n=7). All data is shown as mean ± SEM.

The fact that knockdown of both MEF2A and MEF2D together was required to cause a significant increase in excitatory synapses *in vitro* [5] suggests that MEF2A and MEF2D function redundantly in synaptic development. Therefore, the observed lack of behavioral abnormalities in the *Mef2a* ^KO^ mice may be due to functional redundancy between MEF2A and MEF2D. To examine this possibility, we generated *Mef2a* and *Mef2d* brain-specific double knockout mice (*Mef2a/d* ^DKO^) by crossing *Mef2a* ^KO^ mice with *Mef2d* conditional mice (*Mef2d^loxP/loxP^*) [17]. Mice homozygous for a *Mef2d* null allele are viable and show no obvious phenotypic difference compared to littermate controls (unpublished data). Deletions of both *Mef2a* and *Mef2d* alleles in the brain were confirmed by *in situ* hybridization and quantitative RT-PCR (Fig. 3A,B). *Mef2a/d* ^DKO^ mice were viable to adulthood and had similar weights to their littermates (data not shown). Histological analysis by Hematoxylin and eosin staining revealed no apparent changes in brain morphology compared to wild type littermate CTL mice (data not shown).

To investigate the role of MEF2A and MEF2D in behavior, *Mef2a/d* ^DKO^ mice were subjected to a variety of behavioral paradigms. The *Mef2a/d* ^DKO^ mice exhibited no significant differences in locomotor activity compared to littermate CTL mice (data not shown). In the rotarod test, *Mef2a/d* ^DKO^ mice spent significant less time on the on the rotating rod than the CTL mice suggestive of a deficit in motor coordination (Fig. 3C). On the other hand, *Mef2a/d* ^DKO^ mice showed no apparent behavioral abnormalities in learning and memory when tested for context- and cue-dependent fear conditioning (Fig. 3D). In addition, *Mef2a/d* ^DKO^ mice exhibited no significant differences in anxiety-related behavior or pain sensitivity (data not shown) when compared with CTL mice. These results suggest that MEF2A and MEF2D together play a role in motor coordination, but are not essential for other behaviors including learning and memory.

### Deletion of both MEF2A and MEF2D *in vivo* affects hippocampal short-term synaptic plasticity but does not alter synapse number

To determine whether the development of synapses is affected by the loss of both MEF2A and MEF2D *in vivo*, we investigated dendritic spine density on *Mef2a/d* ^DKO^ hippocampal CA1 pyramidal neurons using Golgi staining. We chose to examine hippocampal CA1 because of the high level of expression of *Mef2a* and *Mef2d* in this subregion of the hippocampus. Surprisingly, the number of spines per 10 µm length of dendrite in pyramidal neurons was not significantly different in *Mef2a/d* ^DKO^ compared to wild type littermate CTL mice (Fig. 3E). To examine whether MEF2A and MEF2D regulate synaptic plasticity, we performed electrophysiological field recordings on hippocampal slices prepared from *Mef2a/d* ^DKO^ and CTL mice. No significant difference between *Mef2a/d* ^DKO^ and CTL mice was observed in hippocampal long-term potentiation (LTP) induced by stimulating the Schaffer collateral pathway using theta-burst stimulation (Fig. 3F). We next examined whether MEF2A and D regulate other key synaptic properties. The field excitatory post-synaptic potential (fEPSP) input-output curves were unchanged in *Mef2a/d* ^DKO^ mice compared to CTL mice (Fig. 3G). However, paired-pulse facilitation (PPF), an index of presynaptic release probability, was increased in *Mef2a/d* ^DKO^ mice, suggesting that MEF2A and MEF2D may act together to regulate neurotransmitter release probability at presynaptic sites (Fig. 3H). These results suggested that the loss of both MEF2A and D does not affect synapse number *in vivo*, or long-term synaptic plasticity, but causes significant alterations in short-term plasticity, consistent with a decrease in presynaptic release probability.

### Deletion of both MEF2A and MEF2D does not impact basal synaptic transmission

**Figure 4:**
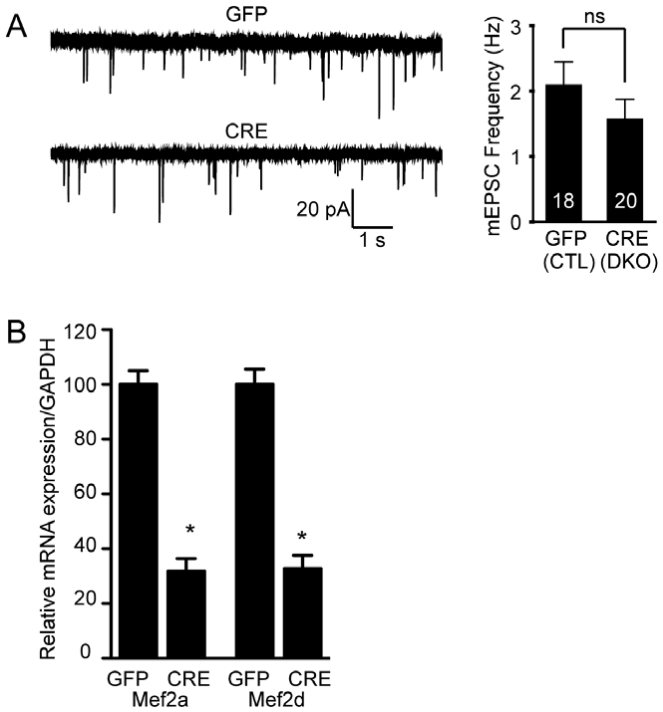
The loss of MEF2A/D does not alter basal synaptic transmission. (**A**) mEPSC frequency is unchanged upon deletion of *Mef2a/d* in hippocampal culture neurons using lentivirus expressing Cre recombinase (p>0.05). The number of recordings is shown in the bar graph for the GFP and Cre infected neurons. Data is shown as mean ± SEM. (**B**) Lentivirus containing either GFP or Cre was infected at 4 DIV in hippocampal culture neurons and cells were harvested at 15–18 DIV, in parallel with the time of recordings. Quantitative PCR was used to confirm the deletion of *Mef2a/d* (*p<0.05). Relative mRNA expression of *Mef2a/d* was normalized to GAPDH (GFP, n=6; Cre, n=6). Data is shown as mean ± SEM.

To more closely investigate the impact of loss of MEF2A and MEF2D function on unitary spontaneous synaptic transmission, we turned to in vitro experiments. We made primary dissociated hippocampal cultures from newborn floxed *Mef2a/d* mice and allowed them to age 4 days *in vitro* before infecting them with high titer lentivirus expressing Cre recombinase tagged with GFP (Cre-GFP), or GFP as a control. One week later, we found no significant difference in the frequency of mEPSCs in high titer Cre-infected floxed *Mef2a/d* neurons compared to GFP-infected neurons, suggesting the selective loss of MEF2A and MEF2D does not impact spontaneous excitatory synaptic transmission (Fig. 4A). This finding also indicates lack of alterations in synapse numbers after loss of MEF2 transcription factor in vitro. Quantitative PCR was used to confirm the deletion of *Mef2a/d* in the cultures (Fig. 4B).

### Deletion of MEF2A, MEF2C and MEF2D increases apoptosis in brain and decreases basal synaptic transmission

**Figure 5:**
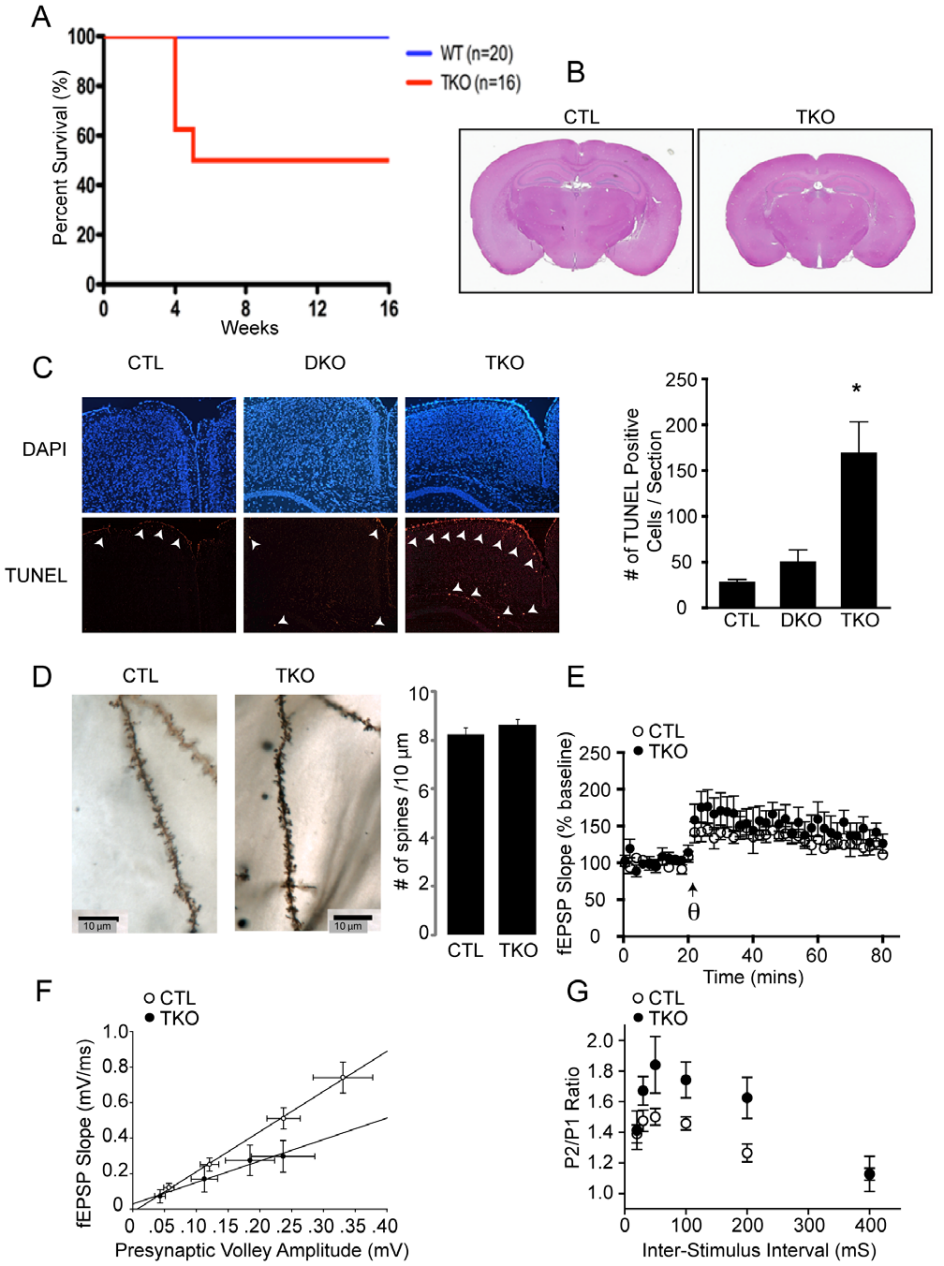
Brain-specific triple deletion of mef2a, c and d impairs survival, brain size and synaptic plasticity. (**A**) Kaplan-Meier survival curves from littermate CTL and *Mef2a/c/d* ^TKO^ (TKO) mice. (**B**) Hematoxylin and eosin staining of coronal section from littermate CTLs and *Mef2a/c/d* ^TKO^. (**C**) Representative TUNEL staining of sections from littermate CTL and *Mef2a/c/d* ^TKO^ brains at 1 month of age. The white arrows indicate TUNEL stained cells. Bar graph shows an increase in apoptotic cells in *Mef2a/c/d* ^TKO^ mice. Data is shown as mean ± SEM. (**D**) Representative images of Golgi-stained CA1 pyramidal neurons from CTL and *Mef2a/c/d* ^TKO^ (TKO) mice (Data pooled from 15 sections/2 CTL mice and from 38 sections/4 *Mef2a/c/d* ^TKO^ mice). (**E**) CA1 LTP induction and maintenance is normal in *Mef2a/c/d* ^TKO^ mice (CTL, n=5; *Mef2a/c/d* ^TKO^, n=6). (**F**) The slope of the input-output functions was significantly lower in *Mef2a/c/d* ^TKO^ mice compared to littermates controls (P<0.05; CTL, n=10; *Mef2a/c/d* ^TKO^, n=7; solid lines indicate lines of best fit). (**G**) Paired-pulse facilitation is significantly increased in *Mef2a/c/d* ^TKO^ mice at Inter-Stimulus Interval 50, 100, 200 and 400 (mS) compared to littermate CTLs (P<0.05; CTL, n=8; *Mef2a/c/d* ^TKO^, n=7).

Mice lacking MEF2A and MEF2D in the developing CNS show no deficits in hippocampal-dependent learning and memory or LTP, but rather only impairments in measurements of short term synaptic plasticity. This, along with previous work from our group demonstrating a specific role for MEF2C in excitatory synapse number, short-term synaptic plasticity, and learning and memory [7], suggests that it is, in fact, the MEF2C isoform, that is important for regulating memory formation and hippocampal synaptic plasticity in mice. MEF2C is highly expressed in brain regions responsible for memory formation, such as the cortex and dentate gyrus. To more closely investigate the role of MEF2C *in vivo*, we generated brain-specific deletions of MEF2A, MEF2C, and MEF2D by breeding the *Mef2a/d* ^DKO^ mice with *Mef2c* ^loxP/loxP^ mice. Triple deletion (*Mef2a/c/d* ^TKO^) resulted in decreased body weight (data not shown) and partial postnatal lethality by 5 weeks of age (Fig. 5A). Histological analysis by hematoxylin and eosin staining of brain sections showed decreased brain size, but no other obvious abnormalities in the morphologies of various brain regions (Fig. 5B). We examined the number of TUNEL positive cells and found in the brains of CTL and DKO mice the positive nuclei extremely sparse, whereas in the TKO mice there appeared to be massive apoptosis. TUNEL staining revealed an approximate 5-fold increase in the levels of apoptosis in *Mef2a/c/d* ^TKO^ mice compared to wild type or *Mef2a/d* ^DKO^ mice, suggesting that MEF2A, C, and D redundantly regulate neuronal survival (Fig. 5C).

The cell death observed in the *Mef2a/c/d* ^TKO^ mice raises a number of complications in terms of behavioral analysis and interpretations. Therefore, we instead focused on determining whether the development of dendritic spines is affected by the loss of MEF2A, MEF2C, and MEF2D *in vivo* on *Mef2a/c/d* ^TKO^ hippocampal CA1 pyramidal neurons using Golgi staining. Surprisingly, the number of spines per 10 µm length of dendrite in pyramidal neurons was not significantly changed in *Mef2a/c/d* ^TKO^ compared to wild type CTL mice, suggesting that at least structurally excitatory synapse number is normal (Fig. 5D).

To investigate synaptic plasticity in *Mef2a/c/d* ^TKO^ mice, we recorded fEPSPs from Schaffer collateral pathway stimulated CA1 pyramidal neurons. The *Mef2a/c/d^TKO^* mice displayed normal LTP (Fig. 5E), but a significantly decreased input-output curve compared to controls (Fig. 5F), suggesting a decrease in the number of functional inputs in the hippocampal CA1 region of these mice. This could be explained by the fact that *Mef2a/c/d* ^TKO^ mice have increased neuronal apoptosis, presumably decreasing the number of presynaptic neurons and/or impairing their functional neurotransmitter output. Indeed, we found an increase in PPF in *Mef2a/c/d* ^TKO^ mice, suggesting a deficit in neurotransmitter release probability (Fig. 5G). These findings were similar to the results seen in the *Mef2a/d* ^DKO^ mice, indicating that both *Mef2a/d* ^DKO^ and *Mef2a/c/d* ^TKO^ mice have deficits in presynaptic neurotransmitter release. Taken together, these results show that triple deletion of MEF2A, MEF2C, and MEF2D impacts neuronal survival and short-term plasticity, but not long-term synaptic plasticity, in the CA1 subregion of the hippocampus.

## Discussion

In this study, we report on the *in vivo* consequences of late embryonic deletion of *Mef2a*, *Mef2a/d* ^DKO^, and *Mef2a/c/d* ^TKO^ selectively in the brain of mice. In the nervous system, MEF2 transcription factors have emerged as regulators of activity-dependent neuronal survival and differentiation [3], [5], [6], [18], [19]. Here, using behavioral, electrophysiological and synapse morphogenesis analysis, we show that the loss of MEF2A and MEF2D in *Mef2a/d* ^DKO^ *in vivo* does not impact learning and memory, long-term potentiation, or synapse number but does affect short-term synaptic plasticity in the CA1 region of the hippocampus. Generating triple MEF2 knockouts we found the *Mef2a/c/d* ^TKO^ mice have dramatically increased apoptosis in the brain, suggesting that MEF2A, C, and D redundantly regulate neuronal survival.

Previous knockdown of MEF2, using a dominant negative MEF2 mutant, was shown to cause apoptosis in cerebellar granule neurons *in vitro* [3]. The brain-specific *Mef2a* ^KO^, *Mef2a/d* ^DKO^, *Mef2a/c/d* ^TKO^ mice generated for this study were useful in determining specific, as well as redundant, roles of MEF2 isoforms in controlling neuronal survival *in vivo*. *Mef2a* ^KO^ and *Mef2a/d* ^DKO^ mice are normal in brain size and do not show evidence of neuronal apoptosis, whereas *Mef2a/c/d* ^TKO^ mice show smaller brain size and increased apoptotic cell death compared to littermate controls. Interestingly, brain-specific deletion of MEF2C alone does not cause any abnormalities in brain size or apoptosis [7], [8], suggesting the deficits in the *Mef2a/c/d* ^TKO^ are the result of functional redundancy of the MEF2 family members to regulate neuronal survival *in vivo*.

In this study, we found that embryonic deletion of *Mef2a* and *Mef2d* in the brain using the *Mef2a/d* ^DKO^ mice impaired motor coordination, but did not affect other behaviors including hippocampal-dependent learning and memory. *Mef2a* and *Mef2d* are highly expressed in the CA1 subregion of the hippocampus, thus we focused our analysis of spine number on CA1 pyramidal neurons, LTP analysis by stimulation of the Schaffer collateral pathway, and synaptic plasticity measurements in this subregion of the hippocampus.

Recent work has suggested that MEF2 is a critical regulator of excitatory synapse number and function. MEF2A and MEF2D have been reported to negatively regulate excitatory synapse number of hippocampal neurons during synaptic development *in vitro* based on RNAi experiments [5]. Surprisingly, the *Mef2a/d* ^DKO^ mice generated in this study had no changes in the density of dendritic spines, a proxy measure of excitatory synapses, of the CA1 neurons using z-series of Golgi-stained tissue. To more closely examine whether this apparent disparity is due to *in vitro* versus *in vivo* differences, we cultured primary hippocampal neurons from floxed *Mef2a/d* mice and then infected them with a high-titer lentivirus expressing Cre recombinase and monitored *Mef2a/d* deletion. We found no significant difference in the frequency of mEPSCs of the *Mef2a/d* null neurons compared to GFP-infected neurons, suggesting that MEF2A and D are not important regulators of excitatory synapse function and likely not regulators of excitatory synapse number.

In contrast to our findings regarding synapse number in the CA1 region of the *Mef2a/d* ^DKO^ mice, the conditional deletion of *Mef2c* in brain suppresses the number of excitatory synapses in the dentate gyrus and regulates basal and evoked synaptic transmission [7], [8]. MEF2C is highly expressed in the dentate gyrus with only low-level expression in other subregions of the hippocampus [2]. Our previous study on the role of MEF2C in vivo, focused on the analysis of synapse number and function in the dentate gyrus. In the present study, we focused instead on the CA1 subregion because of the high level expression of *Mef2a* and *Mef2d* in this region of the hippocampus. The lack of an alteration in synapse number and the *Mef2a/d* ^DKO^ suggests that MEF2A and MEF2D are not key regulators of synapse number. Our current findings that *Mef2a/c/d* ^TKO^ mice have no changes in the density of dendritic spines of the CA1 neurons, is consistent with the low level of expression of MEF2C in this region of the hippocampus, and suggests that in the surviving neurons, at least structurally, synapses are present at normal levels.

To investigate the functional consequences of MEF2A and D loss, we used field potential recordings to activate Schaffer collateral fibers in the CA1 region to assess synaptic connectivity and found that the *Mef2a/d* ^DKO^ mice had no significant change in input-output curve, suggesting that the number of synapses activated by action potential stimulation is not changed. In contrast, the *Mef2a/c/d* ^TKO^ mice have a reduction in the input-output curve. However, this may be due to these mice having a decrease in the number of neurons because of the ongoing apoptosis. We also investigated presynaptic function by measuring PPF, a short-term enhancement of synaptic activity in response to the second of two paired stimuli, believed to be due to residual calcium in the presynaptic terminal after the initial stimulus [20]. The *Mef2a/d* ^DKO^ mice have an increase in PPF suggesting a decrease in release probability, a measure of the likelihood of neurotransmitter release following an action potential and an important determinant of synaptic strength. The *Mef2a/c/d* ^TKO^ mice also show an increase in PPF suggesting a decrease in release probability, but it should be noted that this could also be an indicator of synaptic degeneration triggered by ongoing apoptosis [21].

The cell death observed in the *Mef2a/c/d* ^TKO^ mice raises complications in the evaluation of synaptic electrophysiology. Nevertheless, the similar number of spines, normal LTP, and increase in PPF similar to that observed in the *Mef2a/d* ^DKO^ suggest that there is no additive effect of loss of *Mef2c* in the CA1 region, and that synapses in the *Mef2a/c/d* ^TKO^ mice exhibit normal plasticity following an LTP induction protocol. These data suggest that MEF2A and D play only a subtle role in regulating synaptic function. In contrast, the previous reports on the conditional MEF2C knockouts suggest that this isoform has a significant effect in regulating excitatory synapse structure and function in dentate gyrus. Our data suggests that while the roles of the MEF2 isoforms are distinct in their impact on synaptic number and function, they have overlapping effects in cellular maintenance and survival. Collectively, these results highlight the specific roles for the MEF2A, C and D isoforms in regulation of synaptic function and structure *in vivo*.
